# SCAMMED BY LOVE: THE ROLE OF LONELINESS, TRUST, AND AGE IN FINANCIAL LOSSES FROM U.S. ONLINE ROMANCE SCAMS

**DOI:** 10.1093/geroni/igae098.4273

**Published:** 2024-12-31

**Authors:** Rebecca Cole

**Affiliations:** University of Texas at Rio Grande Valley, Edinburg, Texas, United States

## Abstract

Online romance scam (ORS) victims are popularly characterized as lonely, overly trusting older adults targeted by scammers seeking gifts and money through fake romantic relationships. However, research focuses on middle-aged women, overlooking risks to older adults using social networking, online dating and Internet technologies. The present exploratory research aims to identify these risks by examining the associations among age, feelings of loneliness and trust, and ORS money loss from data collected in a nationwide online survey about U.S. ORS victimization. Male (n = 4) and female (n = 27) participants who were victimized in an ORS and lost any amount of money were identified through community partners and social media. Most participants reported their race as White (n = 17) or Black or African American (n = 10) and had a college degree (n = 22). Their average age was 52 years. The mean reported money lost to the ORS was $133,647. Pearson’s product-moment correlation was used to identify the variables’ associations, and Cohen’s D was used to determine the effect size of the relationships. The only statistically significant result identified a medium-strength relationship between age and ORS money loss r(28) =.584, p <.01. This finding contributes to the limited knowledge in the U.S. of ORS by giving some foundational insight into the role of age and ORS victimization. Technology continues to be a constant in most adults’ lives, and more support is needed to create a safe online Internet experience for all adults.

